# Organ-Specific Diversity of Secoiridoids in *Ligustrum japonicum* Thunb.

**DOI:** 10.3390/molecules31010174

**Published:** 2026-01-02

**Authors:** Sang Won Yeon, Qing Liu, Hak Hyun Lee, Se Jeong Kim, Su Hyeon Lee, Mun-Ock Kim, Bang Yeon Hwang, Mi Kyeong Lee

**Affiliations:** 1 College of Pharmacy, Chungbuk National University, Cheongju 28160, Republic of Korea; sangwon1352@chungbuk.ac.kr (S.W.Y.); leehakhyun1997@chungbuk.ac.kr (H.H.L.); sejeong@chungbuk.ac.kr (S.J.K.); byhwang@chungbuk.ac.kr (B.Y.H.); 2 Food and Pharmacy College, Xuchang University, Xuchang 461000, China; liuqing7115@hotmail.com; 3 Natural Product Research Center, Korea Research Institute of Bioscience and Biotechnology (KRIBB), Cheongju 28116, Republic of Korea; hyeon64a3@daum.net (S.H.L.); mokim@kribb.re.kr (M.-O.K.)

**Keywords:** *Ligustrum japonicum* Thunb., new secoiridoids, plant organs, GNPS molecular networking, new secoiridoids

## Abstract

*Ligustrum japonicum* Thunb. (Oleaceae) has long been valued for the medicinal properties. Its fruits are traditionally utilized, while the leaves and branches are generally discarded after fruit harvest. These aerial parts therefore represent underutilized by-products whose phytochemical profiles remain insufficiently characterized. To elucidate the organ-specific chemical diversity and assess the potential value of these underutilized parts, a comparative analysis of the fruits, leaves, and branches was performed using HPLC–MS/MS combined with GNPS-based molecular networking, with a particular focus on secoiridoids, the characteristic metabolites of the Oleaceae family. This approach revealed substantial overlap as well as distinct variations in secoiridoid profiles among the three plant organs. Chromatographic separation yielded 14 secoiridoid derivatives shared across all organs. In addition, four previously undescribed secoiridoids were isolated and identified through spectroscopic analyses: secoligunosides A (**1**) and B (**2**) from the leaves and secoligunosides C (**3**) and D (**4**) from the branches. Among the major identified secoiridoids, oleuropein (**10**), 8Z-nüezhenide (**17**), and GL-3 (**18**) exhibited weak proliferative activity, showing an approximately 10–20% increase compared to control, on human dermal papilla cells. Collectively, these findings demonstrate that the leaves and branches not only contain key secoiridoids found in the fruits but also harbor unique metabolites, highlighting their value as alternative or complementary medicinal resources. The underutilized parts of *L. japonicum* therefore represent promising sources of natural products and warrant further investigation for future therapeutic applications.

## 1. Introduction

*Ligustrum japonicum* Thunb., commonly known as glossy privet, is a flowering plant of the Oleaceae family distributed widely across East Asia, including Korea, China, and Japan, and now cultivated globally. The fruits are ovoid to ellipsoid drupes that turn dark purple to black upon maturation and contain a single seed. The leaves are opposite, leathery, and elliptic to oblong, with entire margins and a glossy dark-green adaxial surface. The branches are woody, cylindrical, and glabrous, supporting dense foliage and serving as the main structural framework of the plant [1]. Beyond its ornamental value, *L. japonicum* has long been used in traditional medicine, particularly the fruits, which are recognized for their anti-inflammatory, antioxidant, and anticancer properties [2,3,4,5]. The fruits have also been traditionally consumed as a tonic to enhance vitality and are reported to improve hair health and prevent hair graying [2]. In contrast, the leaves and branches have received comparatively little attention and are often discarded after fruit harvest.

Plant metabolites are known to exhibit strong organ-specific patterns driven by tissue-dependent biosynthetic regulation [6,7,8]. While some compounds occur broadly across plant organs, others are localized to specific tissues, reflecting functional specialization and contributing to chemotaxonomic differentiation. Previous phytochemical studies on *L. japonicum* have focused primarily on the fruits, which contain secoiridoids, triterpenes, and phenolic derivatives as major constituents [9,10]. Although several compounds have been reported from the leaves and branches, their phytochemical profile remains incompletely characterized [11].

Given the recognized importance of secoiridoids as signature metabolites within the Oleaceae family and the likelihood of organ-dependent metabolic variation, a comprehensive comparison of *L. japonicum* plant parts is warranted. This study, therefore, aimed to elucidate the secoiridoid diversity across fruits, leaves, and branches through high-performance liquid chromatography–tandem mass spectrometry (HPLC–MS/MS) combined with molecular networking on the Global Natural Products Social (GNPS) platform [12,13]. This integrative approach enabled visualization of secoiridoid-related metabolite relationships and identification of both shared and organ-specific constituents. The findings provide new insights into secoiridoid distribution within *L. japonicum*, highlight underutilized aerial parts, and contribute to the broader chemotaxonomic understanding of the Oleaceae family.

In addition, *L. japonicum* has been traditionally used to promote hair health and prevent hair graying [2], and secoiridoids represent the major class of bioactive constituents in this species. Based on this ethnopharmacological background and chemical profile, human dermal papilla cells were selected as a relevant in vitro model to evaluate the proliferative potential of the identified secoiridoids.

## 2. Results and Discussions

### 2.1. Molecular Networking Analysis of L. japonicum Extracts

**Figure 1:**
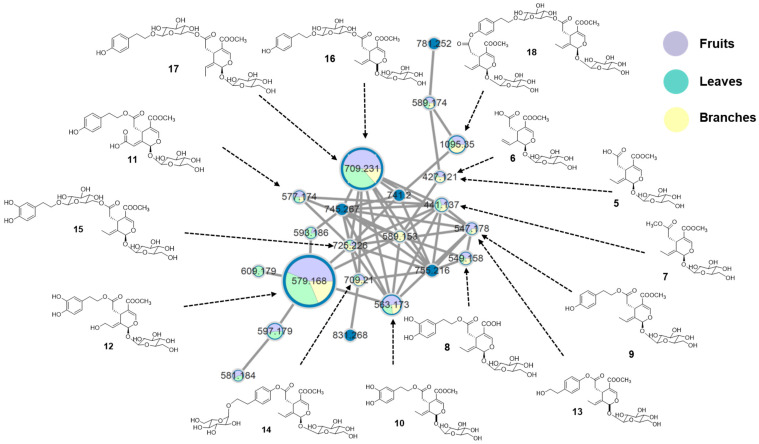
Molecular networking analysis of extracts from *L. japonicum* fruits, leaves, and branches, showing structures matched by the GNPS library.

The three organs of *L. japonicum,* including fruits, leaves, and branches, were initially extracted and the resulting extracts were analyzed using UPLC-HRMS/MS. The resulting MS/MS data were processed through classical molecular networking on the GNPS platform and visualized in Cytoscape. In the constructed network (Figure 1), clusters were color-coded according to plant parts, while LC-MS/MS ion intensities were reflected by node sizes using the “sum (precursor intensity)” function. This comparative visualization revealed two dominant nodes consistently present across all parts. Library matching identified these nodes as 10-hydroxyoleuropein (**12**) and nüezhenide (8*E* or 8*Z*) (**16** and **17**), together forming a conserved secoiridoid core network among the samples.

Several associated clusters containing structurally related metabolites were also observed in all extracts, indicating a broadly shared distribution of core secoiridoids. Subsequent targeted fractionation of selected extracts, combined with MS/MS spectral interpretation and chromatographic isolation, further led to the identification of fourteen known secoiridoid derivatives by comparison with previously reported data [14,15,16,17,18,19,20]. These included oleoside-11-methyl ester (**5**), secoxyloganin (**6**), oleoside dimethyl ester (**7**), demethyloleuropein (**8**), ligustroside (**9**), oleuropein (**10**), oleuropeinic acid (**11**), 10-hydroxyoleuropein (**12**), excelsioside (**13**), excelsioside-O-β-D-glucopyranoside (**14**), neonüzhenide (**15**), 8*E*-nüzhenide (**16**), 8Z-nüzhenide (**17**), and GL-3 (**18**). Collectively, these compounds were distributed across all three plant parts (Figure 1), supporting the presence of a shared secoiridoid backbone throughout *L. japonicum*.

### 2.2. Isolation of New Secoiridoids from L. japonicum

**Figure 2:**
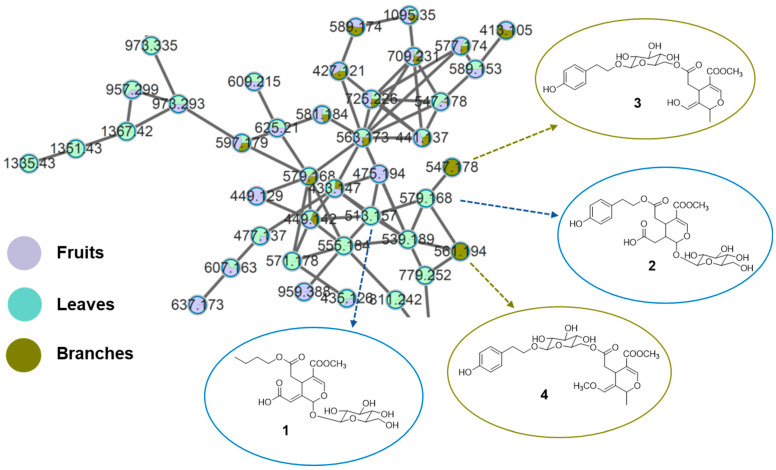
Molecular networking analysis of *n*-BuOH extracts from fruits, leaves and branches of *L. japonicum* with the structures of new compounds identified in this study.

To identify organ-specific metabolites, molecular networking analysis was extended to solvent-partitioned fractions. In particular, analysis of the *n*-BuOH fractions revealed the presence of specialized secoiridoids associated with specific plant organs. In this context, “unique nodes” were defined as molecular features that passed the applied feature filtering and clustering criteria and were detected exclusively or predominantly in a single plant organ, forming distinct nodes or clusters within the molecular network. These nodes were either absent or present only at trace levels in the other plant parts under identical analytical conditions and were therefore considered indicative of organ-dependent chemical specialization. Accordingly, they guided subsequent targeted fractionation and isolation efforts. Consistent with this definition, molecular networking of the *n*-BuOH fractions revealed several unique nodes that showed no significant matches in the GNPS spectral library and were selectively detected in the leaves and branches (Figure 2).

**Figure 3:**
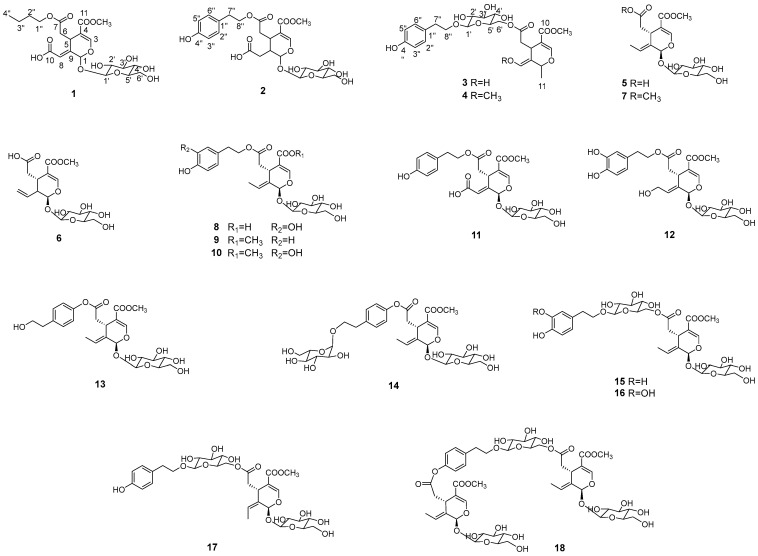
Compounds **1**–**18** isolated from *L. japonicum*.

Guided by these organ-specific features, four previously unreported secoiridoids (**1**–**4**) together with fourteen known ones (**5**–**18**) were isolated from the leaves and branches of *L. japonicum*. Their structures were elucidated using comprehensive spectroscopic analysis (Table 1 and Table 2) and determines as shown in Figure 3.

**Figure 4:**
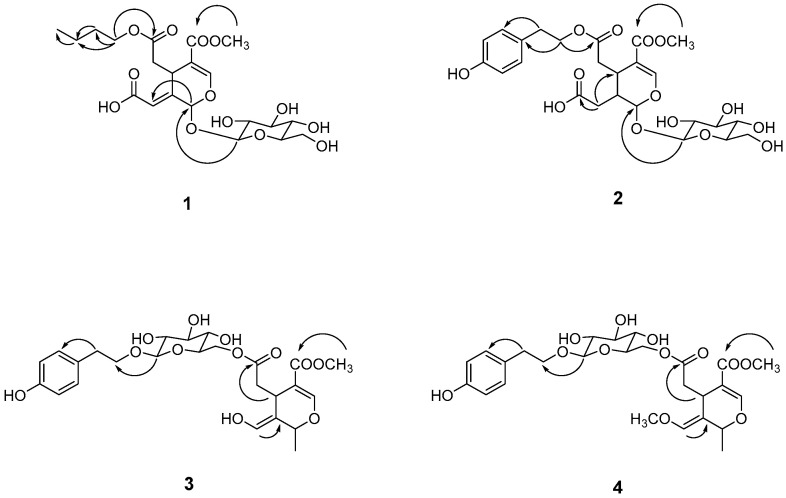
Key HMBC correlations (→) of compounds **1**–**4**.

Compound **1** was isolated as a brown syrup. Its molecular formula was determined to be C_21_H_30_O_13_ from a molecular ion peak at *m*/*z* 513.1577 [M + Na]^+^ (calcd for C_21_H_30_NaO_13_^+^, 513.1579) observed in the HRESI-TOF-MS and supported by the ^13^C NMR spectra. The ^1^H NMR spectrum displayed two olefinic protons at *δ*_H_ 7.58 (1H, s, H-3) and *δ*_H_ 6.39 (1H, brs, H-8), corresponding to carbon signals at *δ*_C_ 153.0 and *δ*_C_ 119.9, respectively, as confirmed by the HSQC spectrum. Additional signals included two methines at [*δ*_H_ 6.09 (1H, s, H-1); *δ*_C_ 93.1] and [*δ*_H_ 2.76 (1H, t, *J* = 3.5 Hz, H-5); *δ*_C_ 31.2], one methylene at [*δ*_H_ 4.76 (2H, overlap, H-6); *δ*_C_ 39.8], and one methoxy group at [*δ*_H_ 3.74 (3H, s); *δ*_C_ 50.4]. The ^13^C NMR spectrum further revealed three carbonyl carbons at [*δ*_C_ 171.9, 166.9, and 157.7], and two quaternary carbons at *δ*_C_ 141.8 and 117.6. The presence of a glucose moiety was confirmed by an anomeric proton signal at *δ*_H_ 4.82 (1H, d, *J* = 7.7 Hz, H-1′) and six glucosyl carbons at *δ*_C_ 99.6, 73.3, 77.0, 70.0, 76.4 and 61.3 (Table 1 and Table 2). These data indicated that compound **1** is a secoiridoid glycoside, a major chemical subclass of this plant [8,9]. The HMBC spectrum (Figure 4) further provided the correlations from OCH_3_ to C-11 and from H-1′ to C-1 established the positions of the methoxyl and glucosyl groups as C-11 and C-11, respectively. The overall structure closely resembled oleoside 11-methyl ester (**5**), except that the methyl group at C-10 was replaced by carbonyl carbon [*δ*_C_ 157.1 (C-10)] in compound **1**. Furthermore, the ^1^H and ^13^C NMR spectra revealed additional peaks corresponding to a butyl group at [*δ*_H_ 4.10 (1H, m, H-1″), 4.04 (1H, m, H-1″), 1.63 (2H, m, H-2″), 1.41 (2H, m, H-3″), 0.97 (3H, t, *J* = 7.2 Hz, H-4″); *δ*_C_ 64.3 (C-1″), 30.4 (C-2″), 18.8 (C-3″), 12.7 (C-4″)], which was confirmed by HSQC analysis. The position of the butyl group was determined to be at C-7 based on the HMBC correlations from H-1″ to C-7. Based on these findings, compound **1** was identified as a new secoiridoid glycoside, as shown and was named secoligunoside A.

Compound **2** was isolated as a brown syrup and its molecular formula was deduced as C_25_H_32_O_14_ from the HRESI-TOF-MS data (*m*/*z* 579.1691 [M + Na]^+^, calcd for C_25_H_32_NaO_14_^+^, 579.1684). The ^1^H and ^13^C NMR spectra indicated that compound **2** possessed the characteristic peaks for a secoiridoid glycoside. In comparison with compound **1**, the olefinic proton signal at H-8 was absent, while signals for a methylene group were observed at [*δ*_H_ 2.35 (1H, m, H-8a), 2.48 (1H, m, H-8b); *δ*_C_ 32.2 (C-8)], showing correlation with a carbonyl group. Furthermore, the presence of tyrosol moiety was suggested by the signals for 1,4-disubstituted aromatic ring at [*δ*_H_ 7.08 (2H, d, *J* = 8.4 Hz, H-2″, 6″), 6.73 (2H, d, *J* = 8.4 Hz, H-3″,5″); *δ*_C_ 128.6 (C-1″), 129.5 (C-2″, 6″), 155.7 (C-4″), 114.9 (C-3″, 5″)] and ethylene signals at [*δ*_H_ 2.85 (2H, m, H-7″); *δ*_C_ 33.7] and [*δ*_H_ 4.21 (2H, m, H-8″); *δ*_C_ 65.5], supported by the HMBC correlation from H-7″ to C-2″ and H-8″ to C-1″. However, signals for a butyl group in compound **1** were not detected. Based on these data, compound **2** was identified as a secoiridoid containing a glucose and a tyrosol moities. The HMBC correlations from H-8″ of the tyrosol to C-7 and from H-1′ of glucose to C-1 confirmed the structure of compound **2** as shown, which was named secoligunoside B.

Compound **3** was isolated as a brown syrup. Its molecular formula was established as C_25_H_32_O_12_ from a molecular ion peak at *m*/*z* 547.1797 [M + Na]^+^ (calcd for C_25_H_32_NaO_12_^+^, 547.1786) observed in the HRESI-TOF-MS and supported by ^13^C NMR data. The ^1^H and ^13^C-NMR spectra of compound **3** were quite similar to those of compound **2**, suggesting that it was a secoiridoid possessing a glucoside and tyrosol moieties. However, compound **3** exhibited spectral features not observed in compound **2**. These included a methyl doublet signal at [*δ*_H_ 1.31 (3H, d, *J* = 6.4 Hz, H-12); *δ*_C_ 19.4 (C-12)] as well as the olefinic signals at [*δ*_H_ 6.55 (1H, s, H-8); *δ*_C_ 137.6 (C-8)], replacing the methylene group in compound **2**. Careful analysis revealed chemical shift changes as a downfield shift in glucose H-6′ from [*δ*_H_ 3.71, 3.82] to [*δ*_H_ 4.16, 4.36] and an upfield shift in the tyrosol H-8″ from [*δ*_H_ 4.21] to [*δ*_H_ 3.71, 3.99]. These shifts suggested the rearrangement in the linkage between moieties. As proposed, HMBC correlations from H-1′ to C-8″ and from H-6′ to C-7 confirm the structural connectivity. Specifically, the tyrosol moiety was attached to the anomeric carbon (C-1′) of the glucose and secoirioid skeleton was linked to position C-6′ of the glucose. Based on these findings, compound **3** was identified as a new secoiridoid glycoside, named secoligunoside C.

Compound **4** was determined to have the molecular formula of C_26_H_32_O_12_ based on a molecular ion peak at *m*/*z* 561.1948 [M + Na]^+^ (calcd for C_26_H_32_NaO_12_^+^, 561.1942) observed in the HRESI-TOF-MS. The ^1^H and ^13^C-NMR spectra of compound **4** were nearly identical to those of compound **4**, with the exception of additional methoxyl signals at [*δ*_H_ 3.22 (3H, s); *δ*_C_ 54.7]. The position of the methoxyl group was assigned to C-8 based on the HMBC correlations from *δ*_H_ 3.22 (3H, s) to *δ*_H_ 139.1 (C-8). Based on these spectral data, the structure of **4** was elucidated as shown and was named secoligunoside D.

### 2.3. Proliferative Effects of Major Secoiridoids on Dermal Papilla Cells

*L. japonicum* has traditionally been used in folk medicine to promote hair health and prevent graying, although scientific evidence supporting these effects remains limited. Nutritional deficiencies within hair follicles are known to contribute to hair loss, and their prevalence continues to rise due to environmental and genetic factors. To explore whether secoiridoids from *L. japonicum* may be associated with its traditional hair-related use, the proliferative effects of major secoiridoids were evaluated using human dermal papilla cells as a preliminary in vitro screening model [21,22]. Five major compounds, oleuropein (**10**), 10-hydroxyoleuropein (**12**), neonüzhenide (**15**), 8Z-nüzhenide (**17**), and GL-3 (**18**), were tested on human dermal papilla cells, and their effects on cell proliferation were assessed. Among these, oleuropein (**10**), 8Z-nüzhenide (**17**), and GL-3 (**18**) exhibited weak proliferative activity, showing an approximately 10–20% increase compared to control, under the present experimental conditions (Table 3). Further mechanistic studies as well as in vivo investigations will be required to clarify the functional relevance of the identified secoiridoids.

### 2.4. Underutilized L. japonicum Parts as Potential Sources of Secoiridoids

Molecular networking analysis revealed that the fruits, leaves, and branches of *L. japonicum* share three major constituents, including 10-hydroxyoleuropein (**12**) and 8*E*-nüezhenide (**16**) and 8*Z*-nüezhenide (**17**). In addition, fourteen known secoiridoids were identified across all three plant organs, demonstrating the presence of a shared secoiridoid throughout the species. These findings are consistent with previous reports identifying secoiridoids as characteristic metabolites of *L. japonicum* fruits and of the Oleaceae family.

Despite this shared chemical framework, distinct organ-specific secoiridoid profiles were observed. Molecular networking identified nodes selectively associated with leaves and branches, which guided the isolation and structural elucidation of four previously undescribed secoiridoids: secoligunosides A (**1**) and B (**2**) from the leaves, and secoligunosides C (**3**) and D (**4**) from the branches. This pronounced organ-dependent variation suggests that the spatial regulation of secoiridoid biosynthesis in *L. japonicum* and that underutilized aerial parts contribute unique structural diversity beyond that of the fruits.

Previous phytochemical studies on *L. japonicum* and related *Ligustrum* species have focused primarily on the fruits [9,10], where secoiridoids are well documented as major constituents. In contrast, the chemical profiles of the leaves and especially the branches have remained largely unexplored. The present study expands existing knowledge by demonstrating that these aerial parts not only share known secoiridoids but also produce unique structural variants. This organ-dependent distribution is consistent with secoiridoid diversity reported in other Oleaceae species and highlights the chemotaxonomic relevance of these metabolites within the family [23,24].

*L. japonicum* has traditionally been used in folk medicine to promote hair health and prevent hair graying, although scientific evidence supporting these effects remains limited. In the present study, major secoiridoids, oleuropein (**10**), 8Z-nüzhenide (**17**), and GL-3 (**18**), exhibited weak proliferative effects on human dermal papilla cells. These results provide preliminary support for the traditional use of *L. japonicum* in hair-related applications.

Taken together, the identification of bioactive secoiridoids and their conserved distribution across fruits, leaves, and branches provides a chemical basis for the traditional hair-related use of *L. japonicum*. Moreover, the presence of both shared and unique secoiridoids in the leaves and branches suggests that these underutilized aerial parts may also contribute to their biological potential.

Although the present study provides new insights into the organ-dependent distribution of secoiridoids in *L. japonicum* and their preliminary biological relevance, further studies are warranted to fully elucidate their functional and practical significance. Comprehensive metabolomic investigations using untargeted and targeted approaches would allow a more detailed comparison of secoiridoid profiles among fruits, leaves, and branches, as well as a quantitative assessment of their relative abundance. In addition, systematic evaluation of secoiridoid extraction yields from different plant parts will be important for assessing their feasibility as alternative or complementary medicinal resources.

From a biological perspective, expanded in vitro studies using additional hair-related cellular models and mechanistic assays would help clarify the molecular basis of the observed proliferative effects. Furthermore, in vivo studies will be necessary to validate the hair health–related potential of the identified secoiridoids and to assess their efficacy and safety in physiologically relevant systems. Collectively, these future investigations will contribute to a more comprehensive understanding of the therapeutic potential of *L. japonicum*, particularly its underutilized aerial parts.

Collectively, these results demonstrate that all parts of *L. japonicum,* including the leaves and branches traditionally regarded as post-harvest by-products, represent valuable sources of structurally diverse and potentially bioactive secoiridoids, supporting their potential utilization as alternative or complementary medicinal resources.

## 3. Materials and Methods

### 3.1. Plant Material

The fruits, leaves, and branches of *L. japonicum*, cultivated in the Republic of Korea, were purchased from an herbal market in Jecheon, Korea. The plant material was taxonomically identified by the committee members of the Herbarium of the College of Pharmacy, where voucher specimens were deposited under the codes CBNU2021-LJF (fruits), CBNU2021-LJL (leaves), and CBNU2021-LJB (branches).

### 3.2. General Experimental Procedure

A Bruker DRX 400 or 500 MHz spectrometer (Bruker-Biospin, Karlsruhe, Germany) was used for the analysis of NMR signals using methanol-*d*_4_ as a solvent. The UV and IR spectra were obtained using Jasco UV-550 (JASCO, Tokyo, Japan) and Perkin–Elmer model LE599 (Perkin–Elmer, Waltham, MA, USA) spectrometer, respectively. HR-ESI-MS and UPLC-MS/MS analyses were performed using an Orbitrap Exploris 120 mass spectrometer coupled to a Vanquish UHPLC system (Thermo Fisher Scientific, Waltham, MA, USA). Semi-preparative HPLC (Waters, Milford, MA, USA) was performed using a Waters 515 HPLC pump with a 996-photodiode array detector, and Waters Empower software (Version 3.8.0) using a Gemini-NX ODS-column (150 × 10.0 mm and 150 × 21.2 mm). Column chromatography procedures were performed using silica gel (200–400 mesh, Fisher Scientific, Waltham, MA, USA) and Sephadex LH-20 (25–100 µm, Pharmacia Fine Chemical Industries Co., Uppsala, Sweden). Thin-layer chromatography (TLC) was performed using aluminum plates precoated with Kieselgel 60 F_254_ (0.25 mm, Merck, Darmstadt, Germany).

### 3.3. Extraction of the Fruits, Leaves, and Branches of L. japonicum

Dried fruits (1.2 kg), leaves (2.0 kg), and branches (350.7 g) of *L. japonicum* were each extracted twice with 80% methanol (MeOH). The extracts were concentrated under reduced pressure to yield methanol extracts of the fruits (320.1 g), leaves (506.2 g), and branches (29.9 g). Each extract was suspended in distilled water and successively partitioned with *n*-hexane, CH_2_Cl_2_, EtOAc, and *n*-BuOH.

### 3.4. UPLC-MS/MS and Molecular Networking

Chromatographic separations were performed on a YMC Triart C18 column (100 × 2.1 mm, 1.9 μm) maintained at 30 °C, with a flow rate of 0.3 mL/min. The mobile phase consisted of water containing 0.1% formic acid (A) and acetonitrile containing 0.1% formic acid (B). The elution was carried out using a linear gradient from 10% to 100% B over 10 min.

Mass spectrometric detection was conducted using an Orbitrap-based high-resolution mass spectrometer operated under the same conditions as those previously established for untargeted metabolomic analysis of *L. japonicum* extracts and *n*-BuOH fractions. Data-dependent MS/MS (DDMS) acquisition mode was employed for tandem mass spectrometry analysis.

Following UPLC–HRMS/MS analysis of the crude extracts and *n*-BuOH fractions, the acquired MS/MS data were processed for molecular networking using the GNPS (Global Natural Products Social Molecular Networking) platform. Raw data files were converted to mzML format using MSConvert. The precursor ion and fragment ion mass tolerances were set to 0.02 Da. Molecular networks were generated by connecting nodes with a cosine similarity score ≥ 0.7 and at least six shared MS/MS fragment ions, with a maximum of ten edges allowed per node. The resulting molecular networks were visualized using Cytoscape software (version 3.8).

### 3.5. Isolation of Compounds

The *n*-BuOH fraction of *L. japonicum* fruits (LJFB, 130.9 g) was chromatographed on HP-20 eluted with a mixture of H_2_O-MeOH (100:0 to 0:100) to obtain six subfractions (LJFB 1–6). Subfraction LJFB2 was subjected to MPLC on RP-silica gel eluted with a mixture of MeOH-H_2_O (90:10 to 0:100 gradient) to obtain six subfractions (LJFB2A-F). Semi-preparative HPLC (MeCN-H_2_O, 25:75) of LJFB2B gave compounds **5**, **12** and **13**. Semi-preparative HPLC (MeCN-H_2_O, 30:70) of LJFB2D gave compounds **6**, **7**, **8** and **18**. Compounds **16** and **17** were isolated from LJFB2F by semi-preparative HPLC eluted with MeCN-H_2_O (17:83). Subfraction LJFB4 was subjected to MPLC on RP-silica gel eluted with a mixture of MeOH-H_2_O (90:10 to 0:100 gradient) yielded seven subfractions (LJFB4A-G). Subfraction LJFB5 was subjected to MPLC on RP-silica gel eluted with a mixture of MeOH-H_2_O (90:10 to 0:100 gradient) yielded five subfractions (LJFB5A-E). Semi-preparative HPLC (MeCN-H_2_O, 35:65) of LJFB5A gave compounds **14** and **15**. Compounds **9**, **10** and **11** were purified from LJFB5E by semi-preparative HPLC eluted with MeCN-H_2_O (20:80).

The *n*-BuOH fraction of *L. japonicum* leaves (LJLB, 305.9 g) was chromatographed on HP-20 eluted with a mixture of H_2_O-MeOH (100:0 to 0:100) to obtain six subfractions (LJLB1–6). Subfraction LJLB2 was subjected to MPLC on RP-silica gel eluted with a mixture of MeOH-H_2_O (90:10 to 0:100 gradient) to obtain five subfractions (LJLB2A-E). Compounds **1** and **2** were isolated from LJLB2C and LJLB2E, respectively, by semi-preparative HPLC eluted with MeCN-H_2_O (20:80).

The *n*-BuOH fraction of *L. japonicum* branches (LJFB, 15.0 g) was chromatographed on HP-20 eluted with a mixture of H_2_O-MeOH (100:0 to 0:100) to obtain six subfractions (LJBB1–6). Subfraction LJBB2 was subjected to MPLC on RP-silica gel eluted with a mixture of MeOH-H_2_O (90:10 to 0:100 gradient) to obtain six subfractions (LJBB2A-F). Semi-preparative HPLC (MeCN-H_2_O, 30:70) of LJBB2E gave compounds **3** and **4**.

Secoligunoside A (**1**) Brown syrup; IR ν_max_ 3387, 1697 cm^−1^; UV (MeOH) λ_max_ 223, 295 nm; HRESI-TOF-MS *m*/*z* 513.1577 ([M + Na]^+^ calcd. for C_21_H_30_NaO_13_^+^ 513.1579); ^1^H NMR (500 MHz, CD_3_OD) and ^13^C NMR (100 MHz, CD_3_OD), see Table 1 and Table 2.

Secoligunoside B (**2**) Brown syrup; IR ν_max_ 3449, 1716 cm^−1^; UV (MeOH) λ_max_ 234, 292 nm; HRESI-TOF-MS *m*/*z* 579.1690 ([M + Na]^+^ calcd. for C_25_H_32_NaO_14_^+^ 579.1684); ^1^H NMR (700 MHz, CD_3_OD) and ^13^C NMR (176 MHz, CD_3_OD), see Table 1 and Table 2.

Secoligunoside C (**3**) Brown syrup; IR ν_max_ 3314, 1652 cm^−1^; UV (MeOH) λ_max_ 224, 296 nm; HRESI-TOF-MS *m*/*z* 547.1797 ([M + Na]^+^ calcd. for C_25_H_32_NaO_12_^+^ 547.1786); ^1^H NMR (400 MHz, CD_3_OD) and ^13^C NMR (176 MHz, CD_3_OD), see Table 1 and Table 2.

Secoligunoside D (**4**) Brown syrup; IR ν_max_ 3415, 1670 cm^−1^; UV (MeOH) λ_max_ 234, 317 nm; HRESI-TOF-MS *m*/*z* 561.1948 ([M + Na]^+^ calcd. for C_26_H_34_NaO_12_^+^ 561.1942); ^1^H NMR (400 MHz, CD_3_OD) and ^13^C NMR (100 MHz, CD_3_OD), see Table 1 and Table 2.

### 3.6. Identification of Structures

The structures of the isolated compounds were elucidated based on spectroscopic analyses, including NMR and MS, and confirmed by comparison with published data. The purity of the compounds was assessed using HPLC and NMR. The NMR spectra of compounds **1**–**4** are provided in the Supplementary Information.

### 3.7. Evaluation of Biological Activity

Primary human hair follicle dermal papilla (DP) cells were obtained from CEFObio (Seoul, Korea) and maintained in CEFOgro-HDP growth medium containing 1% (*v*/*v*) supplement mixture under a humidified atmosphere of 5% CO_2_ at 37 °C. To assess the proliferative effects of isolated secoiridoids, DP cells were seeded in 96-well plates at a density of 1 × 10^3^ cells/well and incubated in CEFOgro-HDP conditioned medium containing 1% supplement mixture for overnight. Cells were then treated with various concentrations of isolated secoiridoids, including extracts and purified compounds, for 48 h. Cell viability was measured by CCK-8 assay (Dojindo, Kamimashiki, Kumamoto, Japan), and absorbance was recorded at 450 nm using a microplate reader (Epoch, Biotek, Winooski, VT, USA). CHIR99021 (Sigma-Aldrich, St. Louis, MO, USA) was used as the positive control.

## 4. Conclusions

In this study, molecular networking analysis revealed major constituents commonly present in the fruits, leaves, and branches of *Ligustrum japonicum*, identified as 10-hydroxyoleuropein (**12**) and nüezhenide 8E (**16**) or nüezhenide 8Z (**17**). Additionally, twelve secoiridoid compounds were identified, isolated, and structurally elucidated. Furthermore, plant organ-specific new metabolites were observed, leading to the isolation and characterization of two secoiridoids, secoligunosides A (**1**) and B (**2**), from the leaves, and two secoiridoids, secoligunosides C (**3**) and D (**4**), from the branches.

Although the fruits of *L. japonicum* have traditionally been used as the primary medicinal material, the present findings demonstrate that the leaves and branches, often regarded as post-harvest by-products, contain comparable core secoiridoids as well as unique structural variants. This suggests that these aerial parts possess therapeutic potential that is complementary to that of the fruits. The organ-dependent distribution of secoiridoids further highlights the leaves and branches as valuable and previously underexplored sources of bioactive compounds.

Collectively, this study expands the chemical and therapeutic landscape of *L. japonicum* by demonstrating that not only the fruits but also the leaves and branches represent promising sources of structurally diverse and potentially bioactive secoiridoids. These findings support the reconsideration of underutilized aerial parts as alternative or complementary medicinal resources and provide a foundation for their future application in functional or therapeutic contexts.

## Figures and Tables

**Table 1:** 1H NMR data of compounds **1**–**4** (CD3OD).

No	1	2	3	4
1	6.09 (s)	5.44 (d, 7.0)	3.82 (m)	3.83 (m)
3	7.58 (s)	7.51 (s)	7.54 (s)	7.55 (s)
5	4.96 *	3.40 *	3.74 (t, 4.7)	3.77 (t, 4.8)
6	2.76 (t, 3.5)	2.52 (2H, m)	2.74 (dd, 14.4, 4.8)	2.68 (dd, 15.2, 5.6)
			2.61 (dd, 14.4, 5.2)	2.55 (dd, 15.2, 4.8)
8	6.39 (brs)	2.50 (m)	6.55 (s)	6.58 (s)
9		2.35 (m), 2.48 (m)		
11			1.31 (3H, d, 6.4)	1.30 (3H, d, 6.4)
1′	4.82 (d, 7.7)	4.69 (d, 7.7)	4.31 (d, 7.8)	4.31 (d, 8.0)
2′	3.42, m	3.25 (m)	3.21 (m)	3.22 *
3′	3.36 *	3.40 *	3.43 (m)	3.40 *
4′	3.36 *	3.40 *	3.40 *	3.40 *
5′	3.43 (m)	3.40 *	3.41 (m)	3.44 (m)
6′	3.99 (m)	3.82 (m)	4.36 (m)	4.38 (dd, 11.6, 2.0)
	3.62 (m)	3.71 (m)	4.16 (dd, 11.6, 5.2)	4.15 (dd, 11.6, 6.0)
1″	4.10 (m), 4.04 (m)			
2″	1.63 (2H, m)	7.08 (d, 8.4)	7.08 (d, 8.4)	7.07 (d, 8.8)
3″	1.41 (2H, m)	6.73 (d, 8.4)	6.71 (d, 8.8)	6.71 (d, 8.8)
4″	0.97 (3H, t, 7.2)			
5″		6.73 (d, 8.4)	6.71 (d, 8.4)	6.71 (d, 8.8)
6″		7.08 (d, 8.4)	7.08 (d, 8.4)	7.07 (d, 8.8)
7″		2.85 (2H, m)	2.85 (2H, m)	2.85 (2H, m)
8″		4.21 (2H, m)	3.99 (m), 3.71 (m)	3.95 (m), 3.71 (m)
8-OCH3				3.22 (3H, s)
10-OCH3			3.72 (3H, s)	3.72 (3H, s)
11-OCH3	3.74 (3H, s)	3.67 (3H, s)	3	4

* overlapping.

**Table 2:** ^13^C NMR data of compounds **1**–**4** (CD_3_OD).

No	1	2	3	4
1	93.1	96.3	76.6	76.7
3	153.0	152.7	151.7	151.6
4	107.6	108.8	108.5	108.8
5	31.2	29.3	27.1	26.4
6	39.8	34.9	39.6	39.9
7	171.9	172.8	171.8	171.6
8	119.9	32.2	137.6	139.1
9	141.8	36.7	120.6	116.9
10	157.7		167.2	167.1
11	166.9	167.4	19.4	17.0
1′	99.6	99.1	103.1	103.0
2′	73.3	73.2	73.6	73.6
3′	77.0	77.0	73.8	76.5
4′	70.0	70.1	70.1	70.2
5′	76.4	76.4	76.5	73.9
6′	61.3	61.3	63.3	63.5
1″	64.3	128.6	129.3	129.3
2″	30.2	129.5	129.5	129.5
3″	18.7	114.9	114.8	114.8
4″	12.6	155.7	155.4	155.4
5″		114.9	114.8	114.8
6″		129.5	129.5	129.5
7″		33.7	35.0	35.0
8″		65.5	70.9	70.8
8-OCH3				54.7
10-OCH3			50.7	50.7
11-OCH3	50.4	50.4	3	4

**Table 3:** Effects of major secoiridoids of *L. japonicum* on the proliferation of dermal papilla cells.

Compounds	Proliferation (%)		
	60 μM	40 μM	20 μM
Control		100.0 ± 6.3	
Oleuropein (**10**)	125.2 ± 7.3	109.4 ± 5.6	107.8 ± 7.1
10-Hydroxyoleuropein (**12**)	98.9 ± 9.1	100.1 ± 8.0	99.0 ± 8.4
Neonüezhenide (**15**)	99.8 ± 5.2	98.3 ± 4.5	99.5 ± 1.4
8Z-Nüezhenide (**17**)	118.9 ± 9.8	115.4 ± 7.6	116.3 ± 8.9
GL-3 (**18**) CHIR99021 (2.5 μM) ^(a)^	115.7 ± 7.9	114.1 ± 7.3 158.8 ± 3.7	101.8 ± 3.8

^(a)^ CHIR99021 was used as the positive control.

## Data Availability

The data will be made available on request.

## Supplementary Materials

The following supporting information can be downloaded at: https://www.mdpi.com/article/10.3390/molecules31010174/s1, Figure S1: ^1^H NMR spectrum of compound **1**, Figure S2: ^13^C NMR spectrum of compound **1**, Figure S3: HSQC spectrum of compound **1**, Figure S4: HMBC spectrum of compound **1**, Figure S5: MS NMR spectrum of compound **1**, Figure S6: ^1^H NMR spectrum of compound **2**, Figure S7: ^13^C NMR spectrum of compound **2**, Figure S8: HSQC spectrum of compound **2**, Figure S9: HMBC spectrum of compound **2**, Figure S10: MS NMR spectrum of compound **2**, Figure S11: ^1^H NMR spectrum of compound **3**, Figure S12: ^13^C NMR spectrum of compound **3**, Figure S13: HSQC spectrum of compound **3**, Figure S14: HMBC spectrum of compound **3**, Figure S15: MS NMR spectrum of compound **3**, Figure S16: ^1^H NMR spectrum of compound **4**, Figure S17: ^13^C NMR spectrum of compound **4**, Figure S18: HSQC spectrum of compound **4**, Figure S19: HMBC spectrum of compound **4**, Figure S20: MS NMR spectrum of compound **4**.

## Abbreviations

The following abbreviations are used in this manuscript:

HPLC–MS/MS	High-performance liquid chromatography–tandem mass spectrometry
GNPS	Directory of open access journals Global Natural Products Social
DP	Dermal papilla
