# Supplementary material for: Organ-Specific Diversity of Secoiridoids in Ligustrum japonicum Thunb
Source: Molecules. 2026 Jan 2;31(1):174. doi: 10.3390/molecules31010174 (PMC12787287; doi:10.3390/molecules31010174)
Supplement: Supplementary file 1 [file molecules-31-00174-s001.zip › molecules-4036413-supplementary.pdf]

Diverse secoiridoids of *Ligustrum japonicum* across plant parts

Sang Won Yeon [1, †], Qing Liu [2, †], Hak Hyun Lee [1], Se Jeong Kim [1], Su Hyeon Lee [3], Mun-Ock Kim [3], Bang Yeon Hwang [1], and Mi Kyeong Lee [1,*]

[1] *College of Pharmacy, Chungbuk National University, Cheongju 28160, Republic of Korea*

[2] *Food and Pharmacy College, Xuchang University, Xuchang 461000, China*

[3] *Natural Product Research Center, Korea Research Institute of Bioscience and Biotechnology (KRIBB), Cheongju 28116, Republic of Korea*

[*] Correspondence to:

College of Pharmacy, Chungbuk National University, Cheongju 28160, Korea.

E-mail: mklee@chungbuk.ac.kr (M.K.Lee)

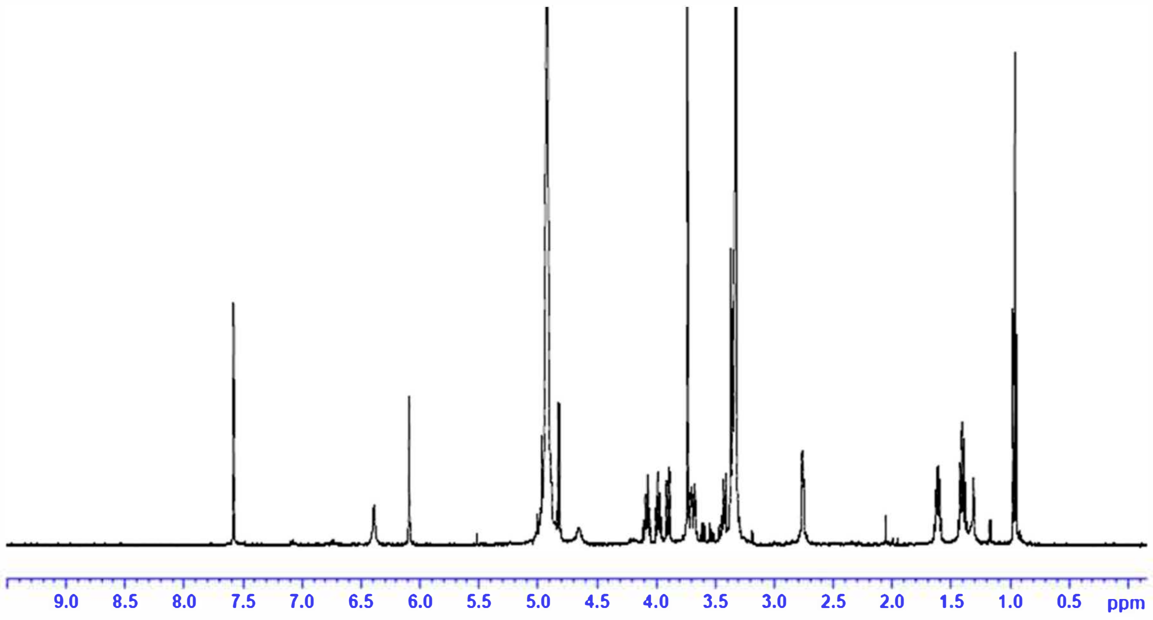

Fig. S1 ^{1}H NMR spectrum of compound **1**

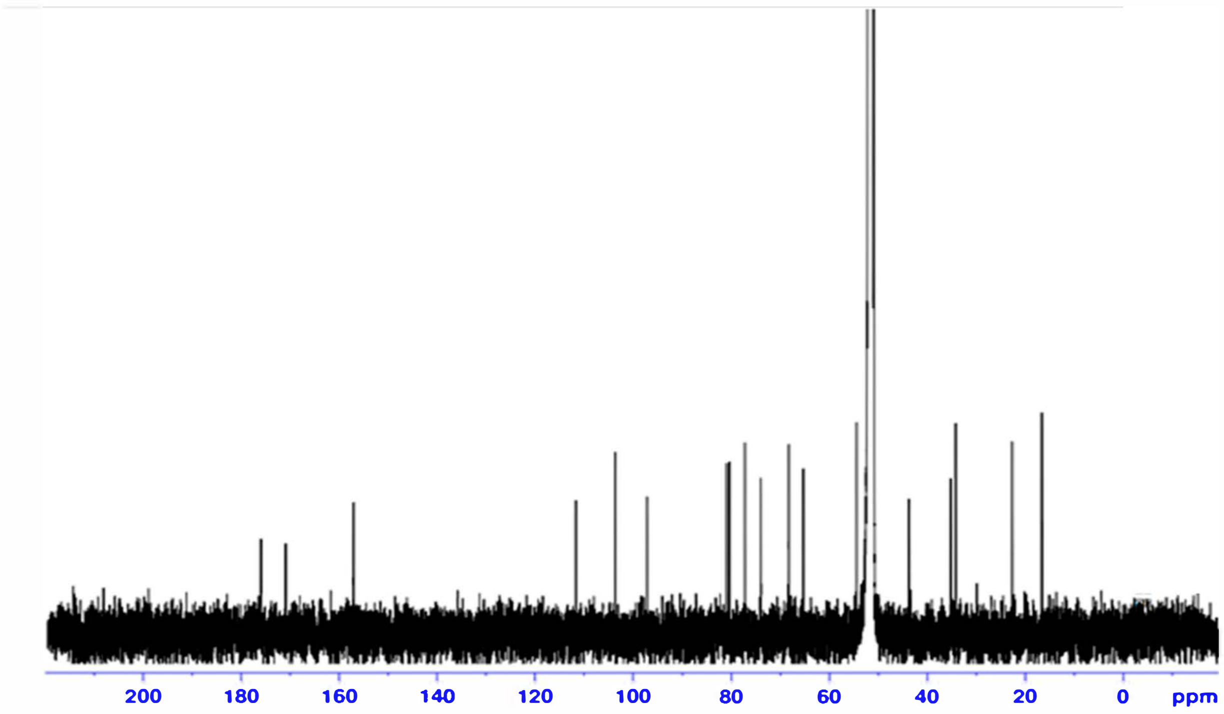

Fig. S2 ^{1}H NMR spectrum of compound **1**

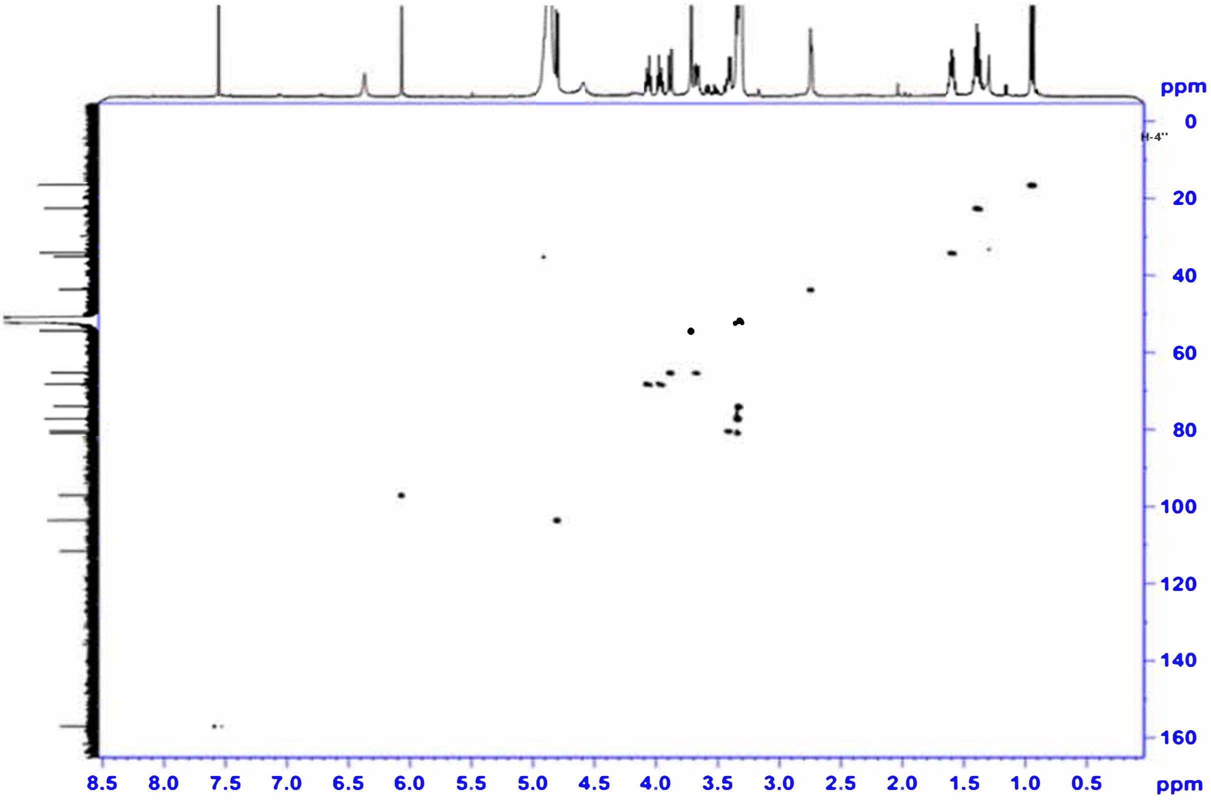

Fig. S3 HSQC spectrum of compound **1**

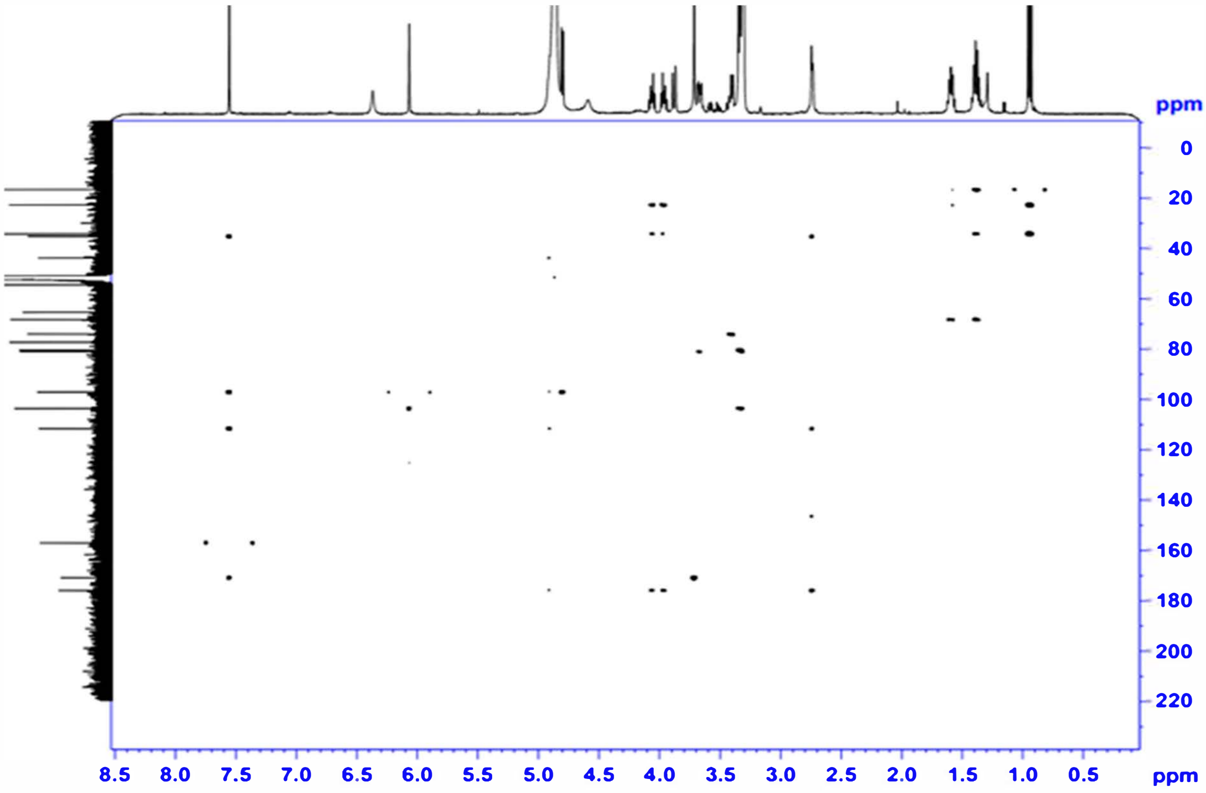

Fig. S4 HMBC spectrum of compound **1**

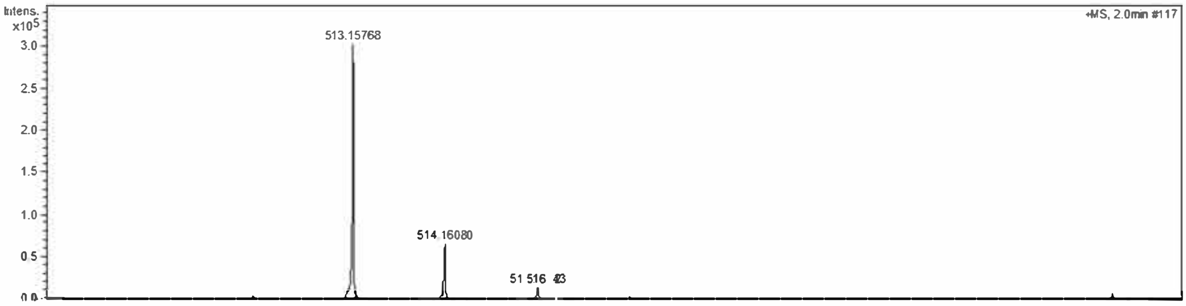

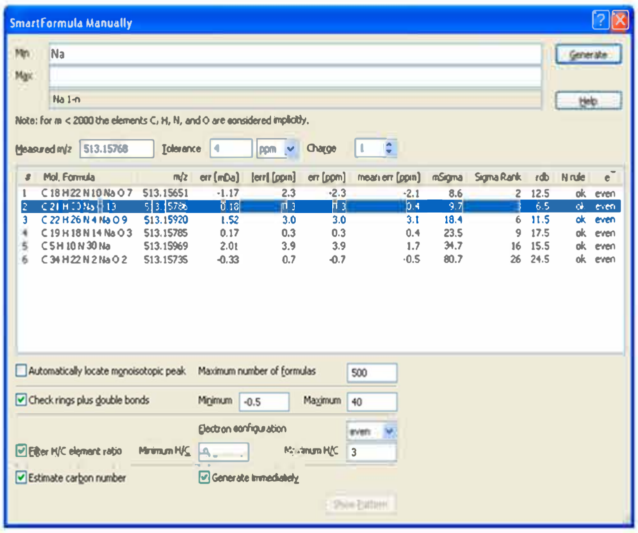

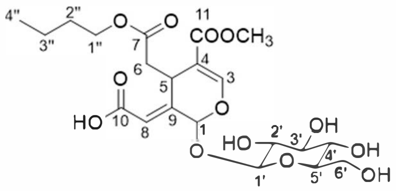

Chemical Formula: $C_{21}H_{30}O_{13}$
Exact Mass: 490.1686

HRESI-TOF-MS

m/z **513.1579**
(calcd. For $C_{21}H_{30}NaO_{13}$)

Fig. S5 MS spectrum of compound **1**

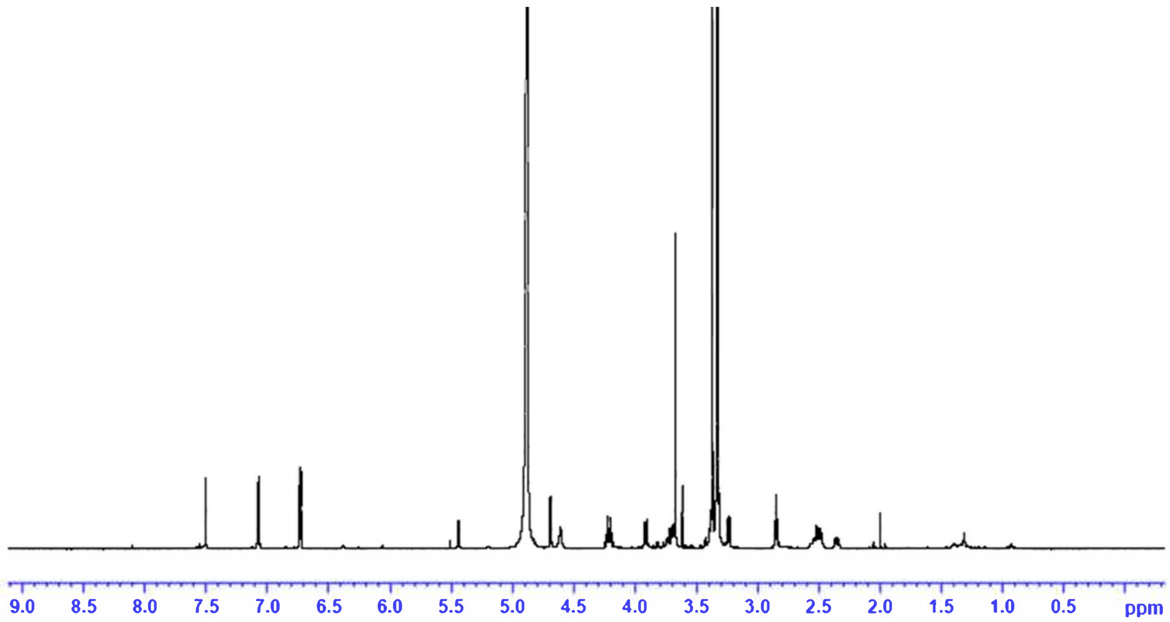

Fig. S6 ¹H NMR spectrum of compound **2**

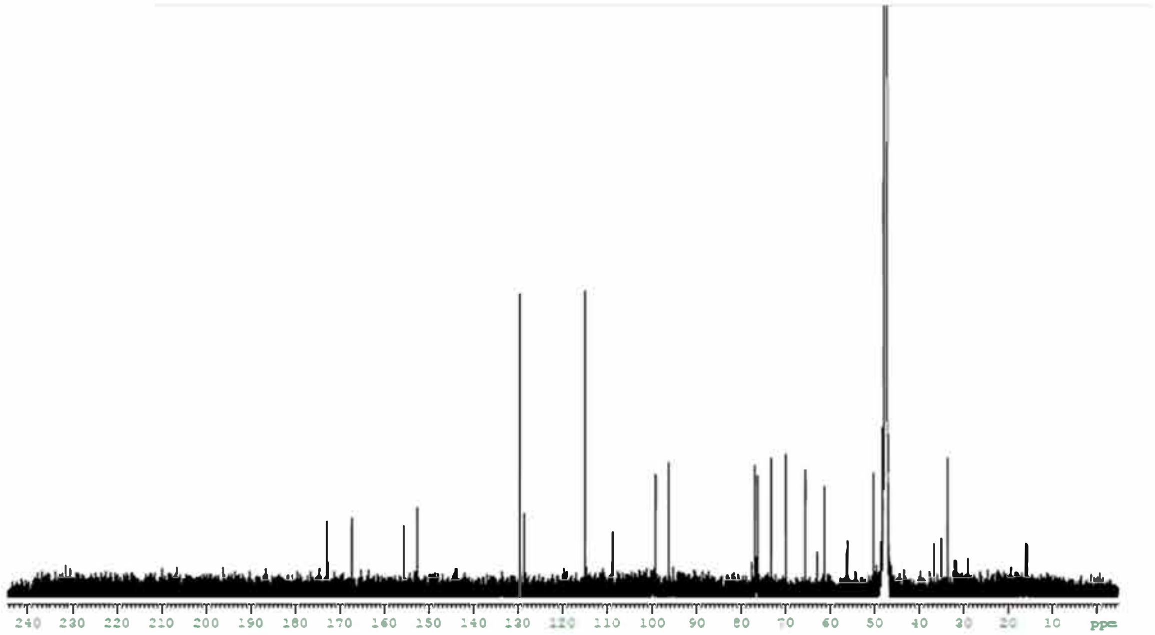

Fig. S7 ¹H NMR spectrum of compound **2**

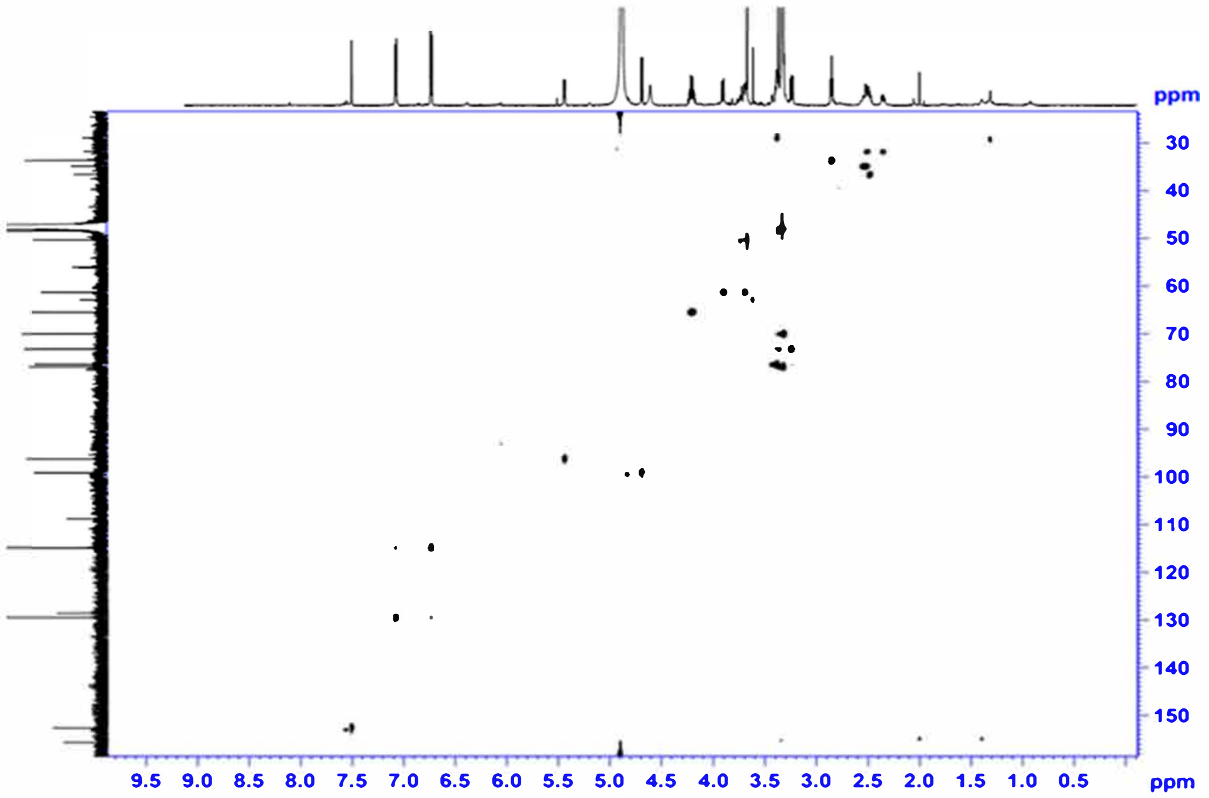

Fig. S8 HSQC spectrum of compound **2**

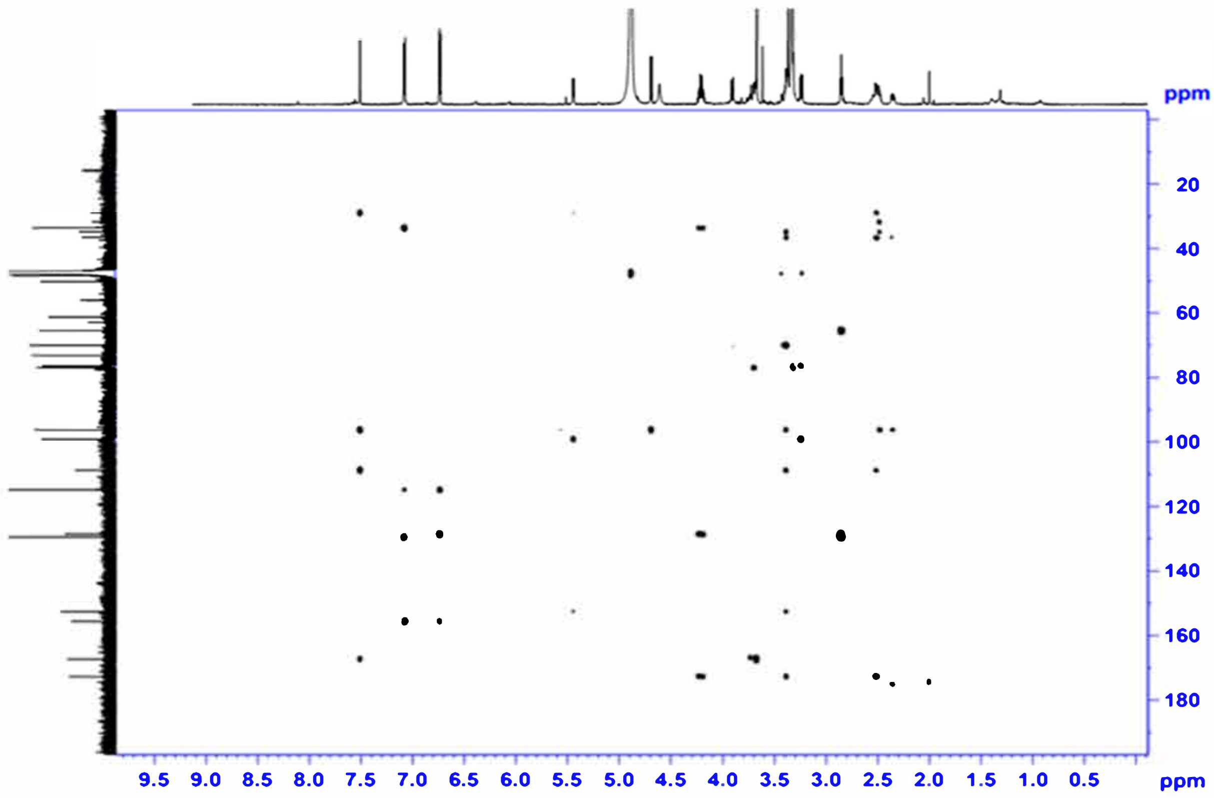

Fig. S9 HMBC spectrum of compound **2**

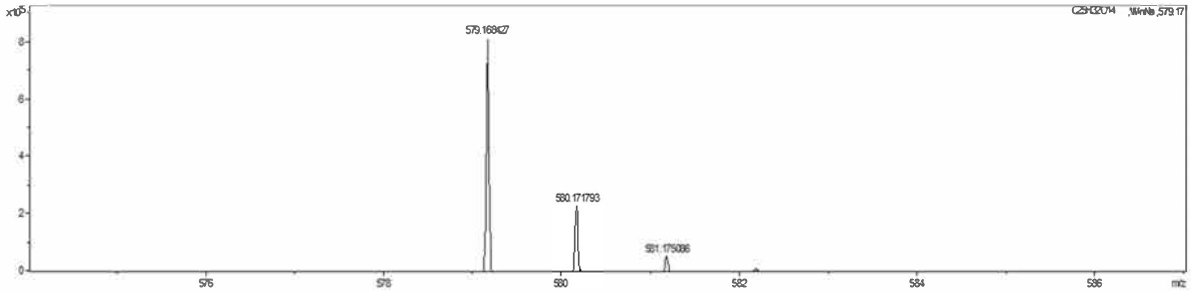

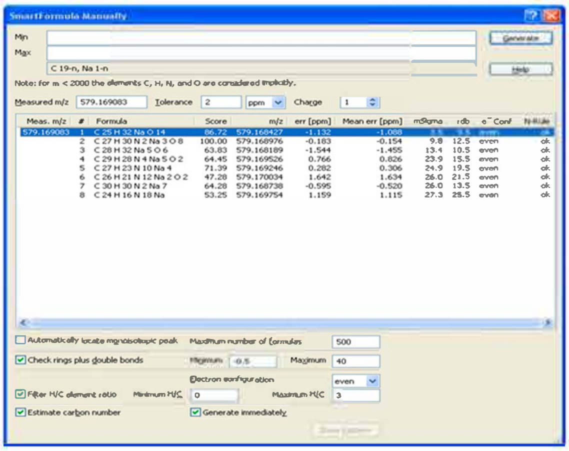

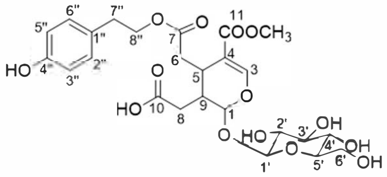

Chemical Formula: $C_{25}H_{32}O_{14}$
Exact Mass: 556.1792

HRESI-TOF-MS

m/z **579.1684**
(calcd. For $C_{25}H_{32}NaO_{14}$)

Fig. S10 MS spectrum of compound **2**

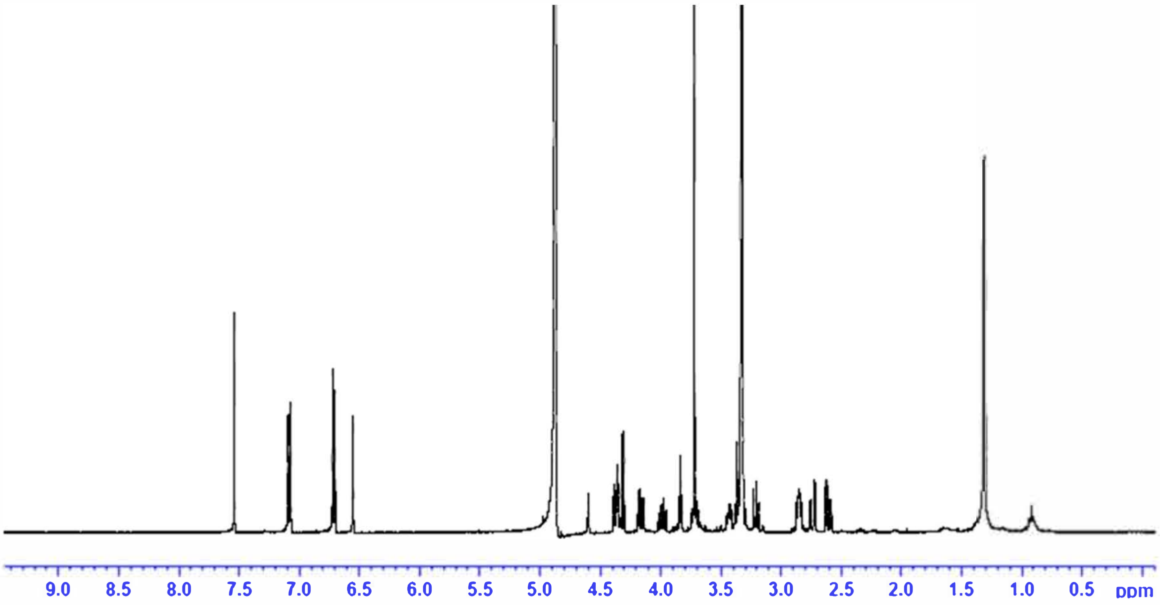

Fig. S11 ¹H NMR spectrum of compound **3**

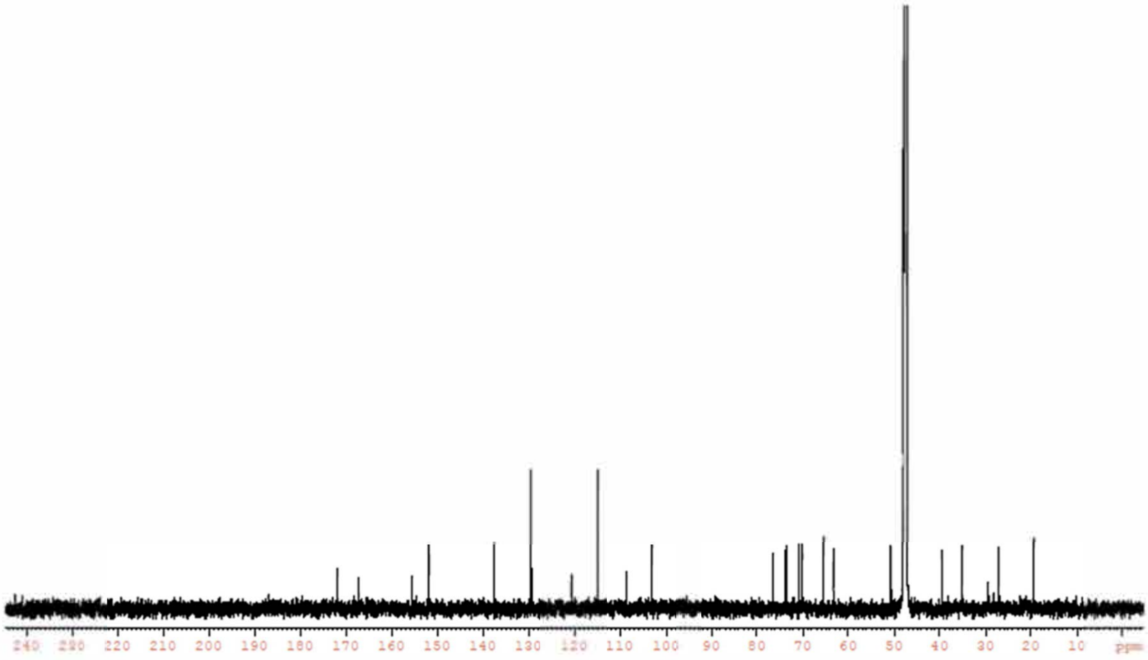

Fig. S12 ¹H NMR spectrum of compound **3**

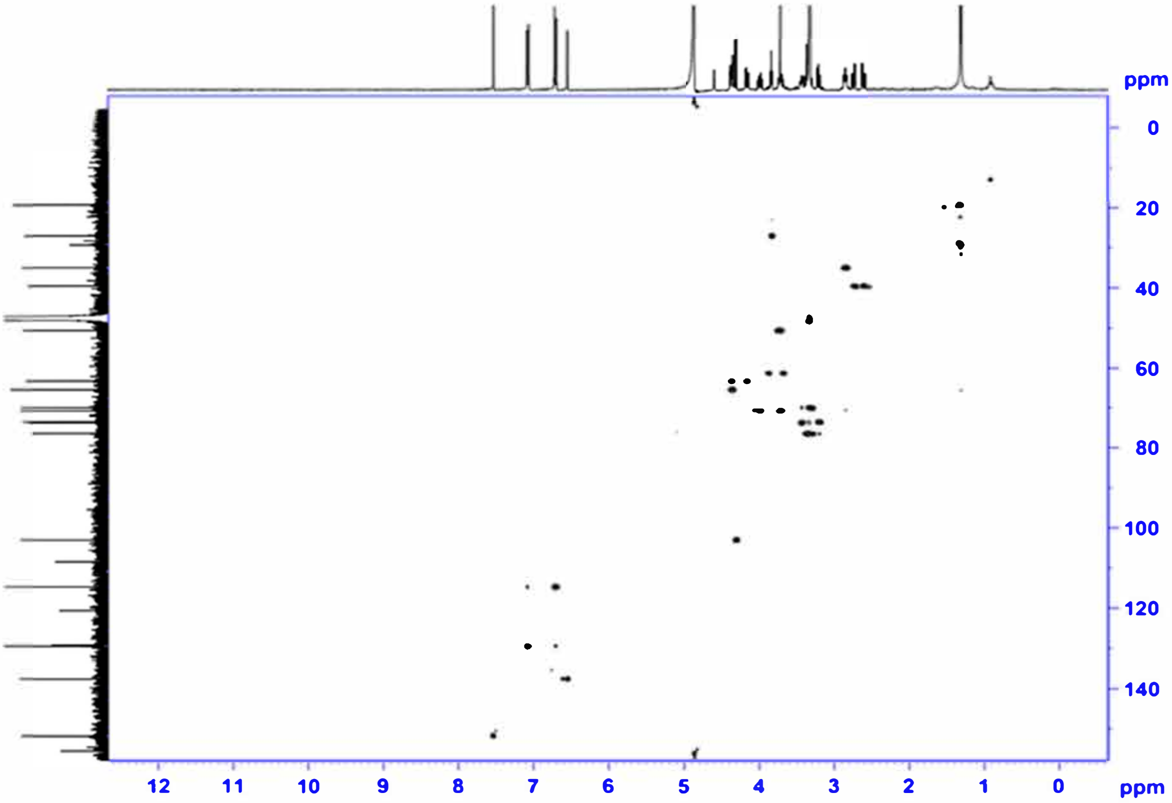

Fig. S13 HSQC spectrum of compound **3**

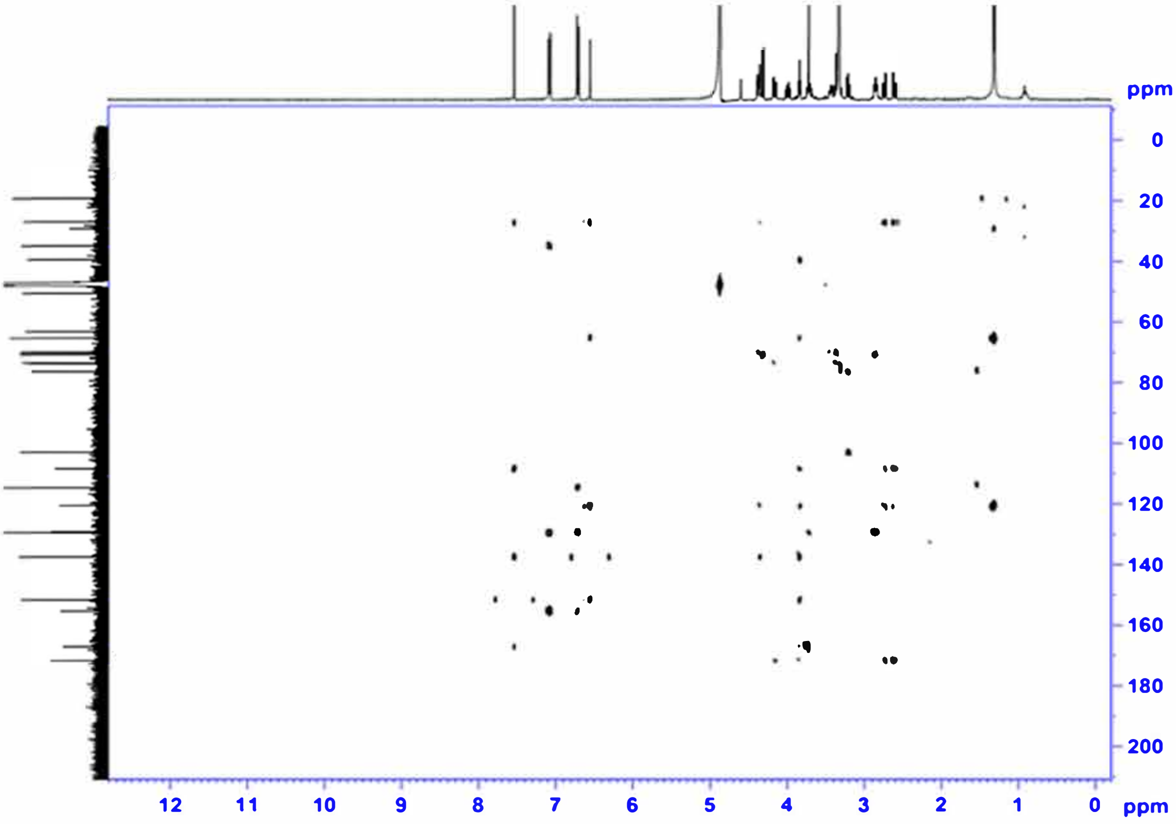

Fig. S14 HMBC spectrum of compound **3**

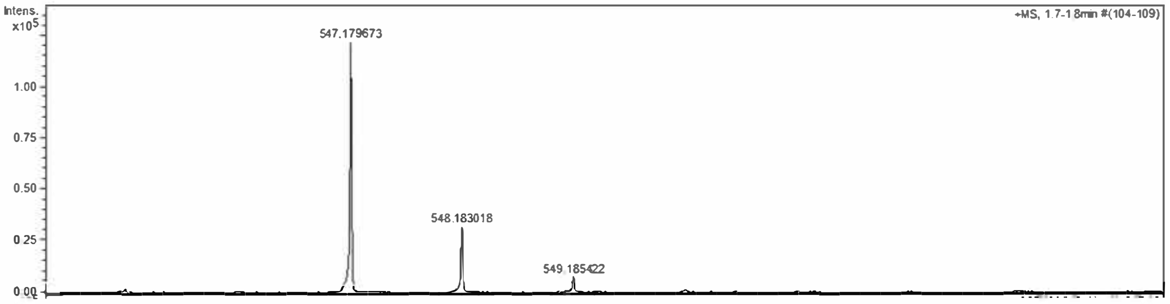

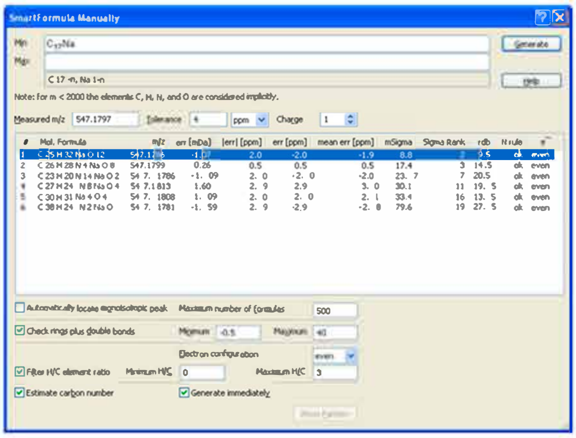

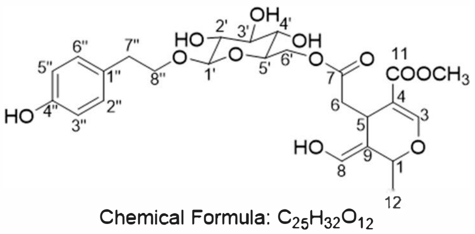

Chemical Formula: $C_{25}H_{32}O_{12}$
Exact Mass: 524.1894

HRESI-TOF-MS

m/z **547.1786**
(calcd. For $C_{25}H_{32}NaO_{12}$)

Fig. S15 MS spectrum of compound **3**

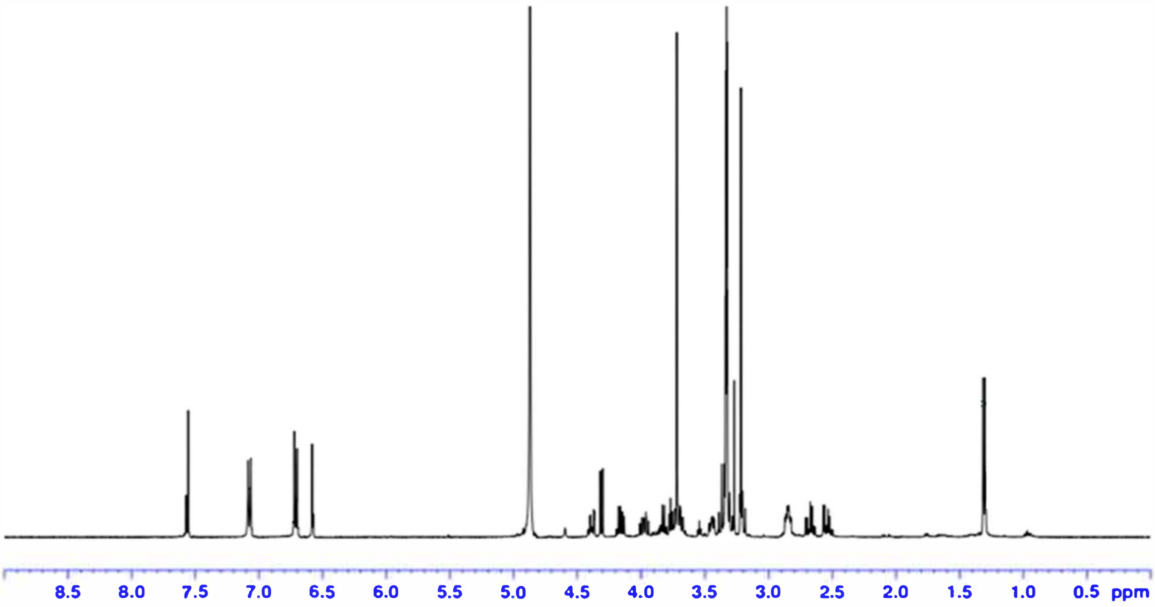

Fig. S16 ¹H NMR spectrum of compound **4**

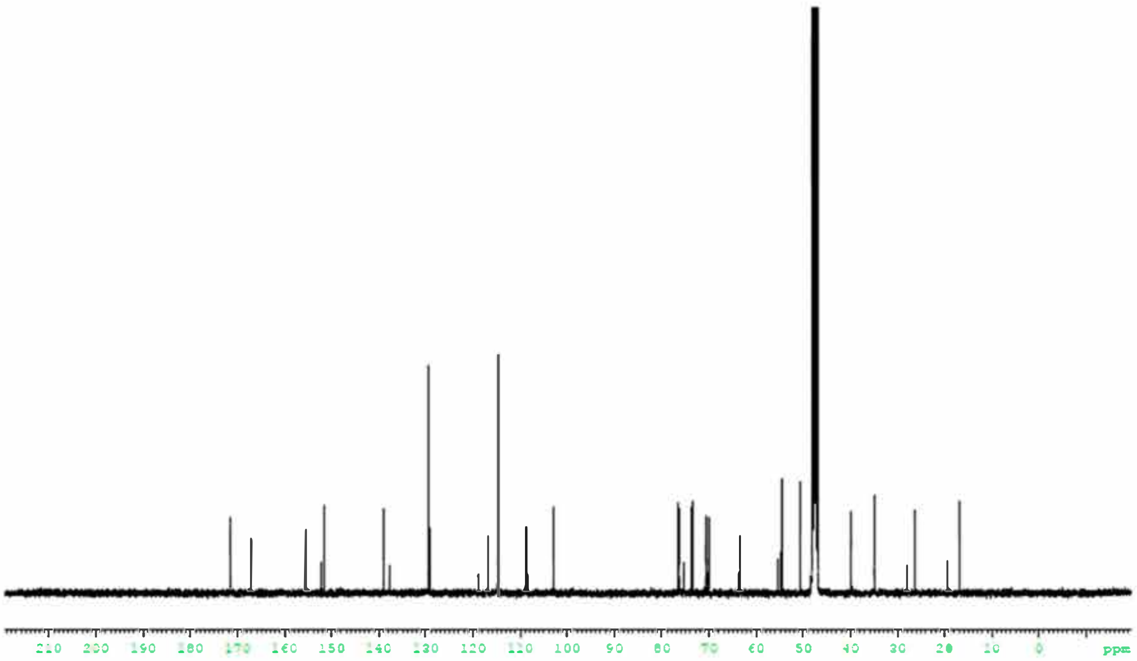

Fig. S17 ¹H NMR spectrum of compound **4**

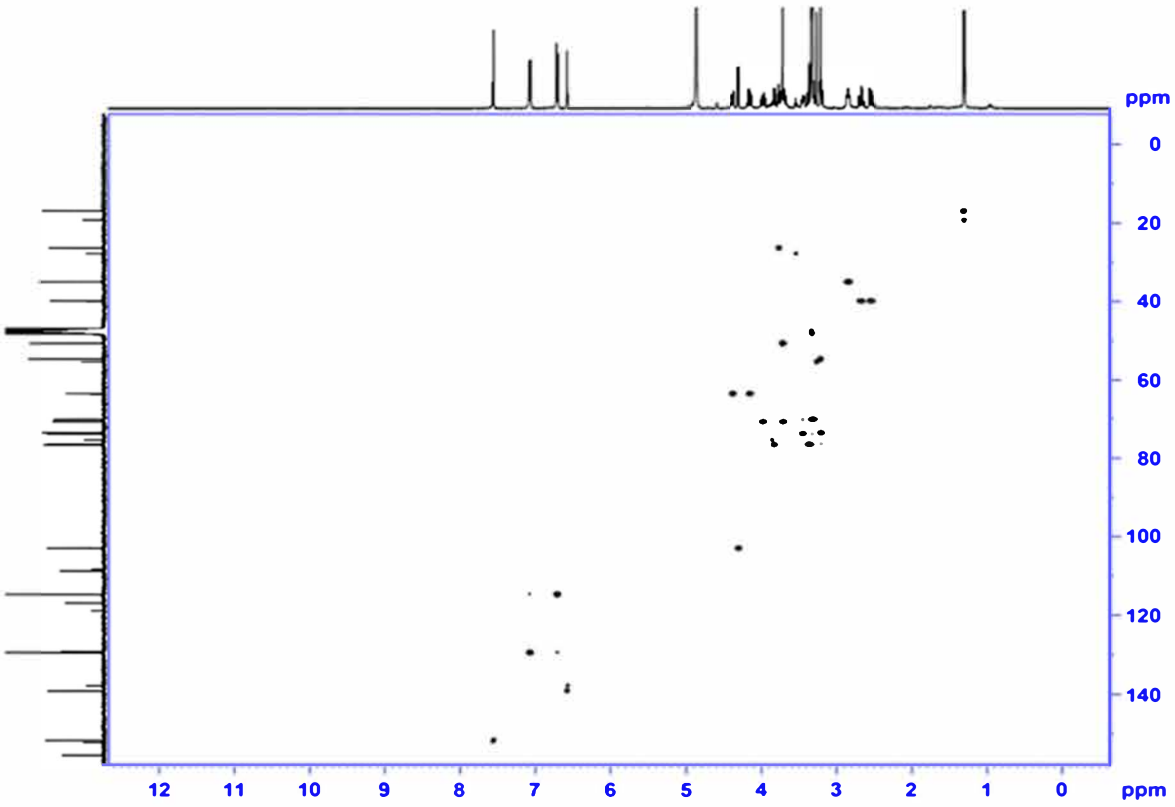

Fig. S18 HSQC spectrum of compound **4**

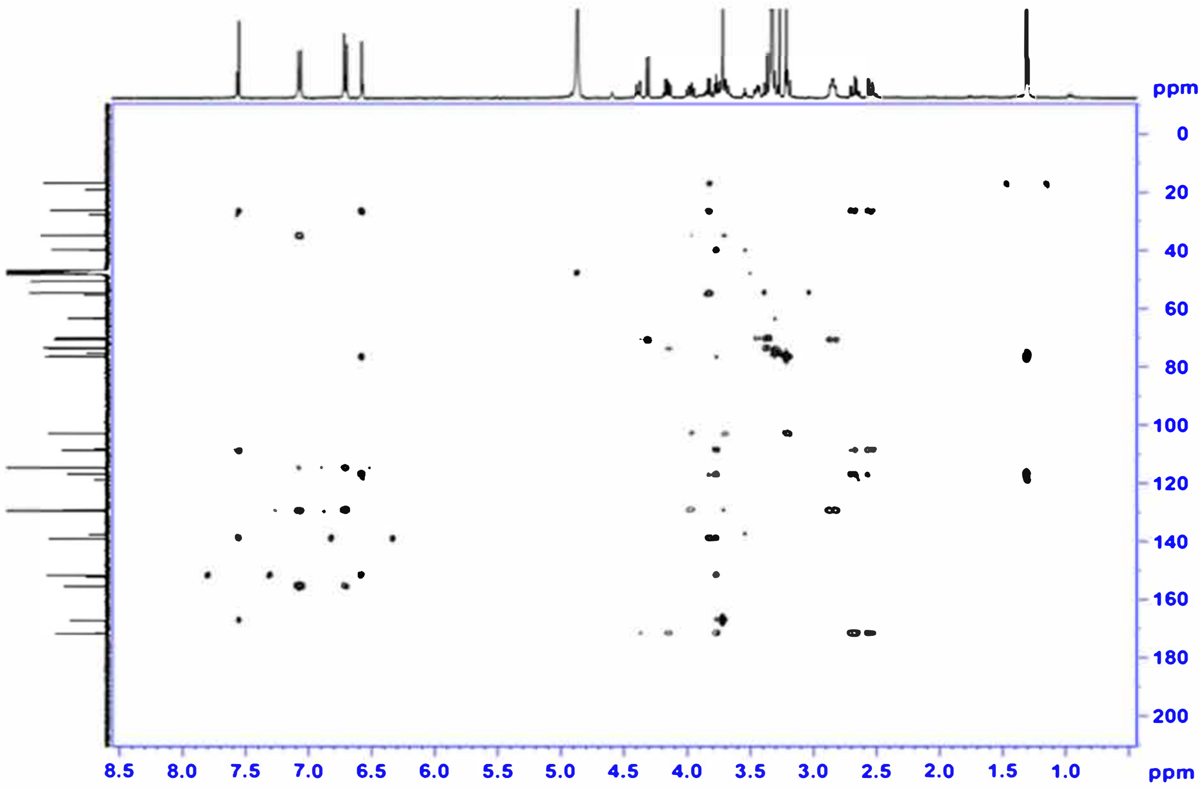

Fig. S19 HMBC spectrum of compound **4**

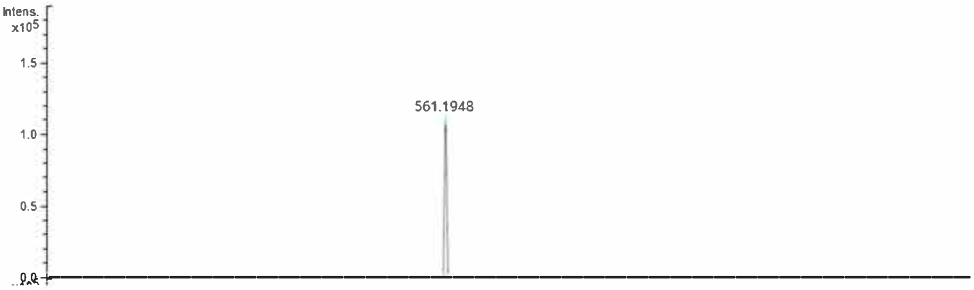

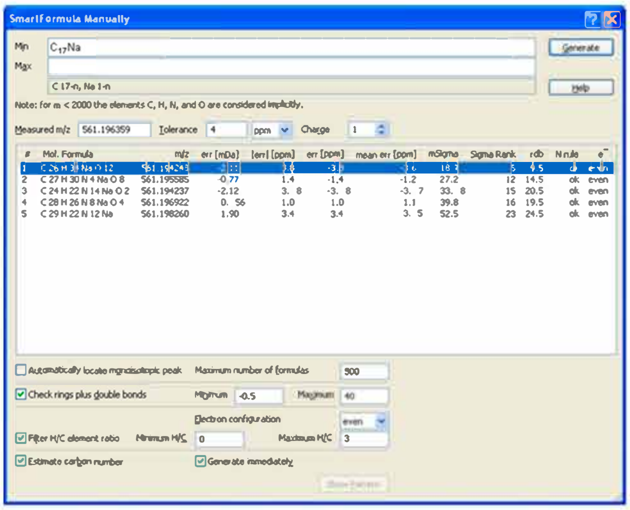

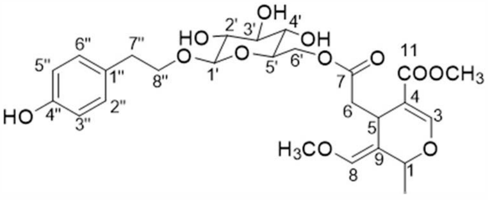

Chemical Formula: $C_{26}H_{34}O_{12}$
Exact Mass: 538.2050

HRESI-TOF-MS

m/z **561.1942**
(calcd. For $C_{26}H_{34}NaO_{12}$)

Fig. S20 MS spectrum of compound **4**
